# A Comparison of the Percentage of “Yes” (Agree) Responses and Importance of Attributes (Constructs) Determined Using Check-All-That-Apply and Check-All-Statements (Yes/No) Question Formats in Five Countries

**DOI:** 10.3390/foods9111566

**Published:** 2020-10-28

**Authors:** Denis Richard Seninde, Edgar Chambers

**Affiliations:** Center for Sensory Analysis and Consumer Behavior, Kansas State University, Manhattan, KS 66502, USA; seninde@ksu.edu

**Keywords:** Check All That Apply, Check All Statements, yes/no, CATA, survey, sensory, marketing, questionnaire

## Abstract

Check All That Apply (CATA) has become a popular type of questionnaire response in sensory/consumer research in recent years. However, some authors have pointed out potential problems with the method. An online survey using either a Check-All-That-Apply (CATA) or Check-All-Statements (CAS) format for questions was conducted to provide a deeper understanding of the response data using the two question formats. With CATA, respondents select all terms or statements that apply from a given list, while, with CAS, respondents must respond (e.g., yes/no or agree/disagree) to each term or statement to show that it applies or does not apply. Respondents from five countries (Brazil, China, India, Spain, and the USA) were randomly assigned one of the two question formats (*N* = 200 per country per method). Motivations for eating items that belong to five food groups (starchy, protein, dairy, fruits, and desserts) were assessed. Results showed that CAS had higher percentages of “agree” responses than CATA. Also, the response ratio of CAS and CATA data was different, suggesting that interpretations of the data from each response type would also be different. Respondents in the USA, China, and Spain took longer to complete the CAS questionnaire, while respondents in Brazil and India had similar time durations for the two question formats. Overall, the CATA format was liked slightly more than the CAS format and fewer respondents dropped out of the survey when using the CATA response type. These findings suggest that the CATA format is quick and relatively easy for consumers to complete. However, it provokes fewer “apply” responses, which some psychologists suggest underestimates applicable terms or statements and CATA provides a different interpretation of data than the CAS format that requires consumers to respond to each term or statement. Further, CAS may overestimate the applicable terms. Consumer insights collected using CATA and CAS can lead to different decisions due to differences in data interpretation by researchers (e.g., marketers, nutritionists, product developers, and sensory scientists). More investigation is needed for the CATA and CAS question formats.

## 1. Introduction

The use of the Check-All-That-Apply (CATA) or the Mark-All-That-Apply question format as reported by Sudman and Bradburn [1] has become popular in consumer research [2,3,4,5,6,7,8,9,10,11,12,13]. This question format asks respondents to check all items that apply from a list of options. For the Check-All-Statements (CAS) or the forced-choice question format, also known as the yes/no format or sometimes called “applicability scoring”, a respondent is asked to check a “yes” or “no” option (or something similar such as agree or disagree) for each item [10,11,14]. Both the CATA and CAS question formats have been used extensively for questionnaires that are designed to be completed by respondents with little or no intervention. Although the research literature tends to be fairly recent, multiple industries have been using this procedure at least since the early 1980s [1,15]. 

CATA was developed to reduce the fatigue of respondents while they completed a self-administered questionnaire. This tool provides an easy and non-tedious way of collecting multiple responses that are reproducible [3,8,16,17,18]. However, the CATA format has been criticized for ambiguity in interpreting the absence of a checkmark on listed options. The unchecked items can be interpreted as those that were not applicable or those for which the respondent was uncertain about the applicability. It is also possible that the respondent did not notice the item(s) as they hurriedly read the list of items [5,10] or that they only paid attention to the first items or a limited set of items to save time. Survey research theory [19] suggests that for self-administered surveys (e.g., online surveys), respondents may select the first acceptable response(s) and not pay attention to later responses because it takes too much effort. This may particularly be true based on the cognitive elaboration model [20] when the respondent is in visual mode (e.g., reading responses) because the respondent takes more time to consider the first options. Ares and Jaeger [21] showed that the order in which items in the CATA question were presented had an impact on the results. For instance, items that appeared in the top left corner of the ballot were checked more frequently as compared to items that were placed at the bottom of the ballot and this consequently affected the total number of responses provided. This suggests that the items seen early in the questionnaire are more likely to be rated as “apply” than those later in the questionnaire. Simply randomizing the terms can reduce the impact of order bias on specific terms, but does not eliminate the problem and exacerbates the impact that order bias has on differences in scoring frequency when attempting to compare consumers or cluster them based on their responses. In a recent study with children, different response patterns were found, suggesting that cognitive impacts are apparent even in CATA questionnaires [22]. 

Although some researchers have shown that both the CAS and CATA question formats provide similar results in terms of outcomes, time, and survey satisfaction, other researchers [12,23] disagree and suggest otherwise. Fundamentally, the CAS seeks a response (e.g., yes or no) for each item, while the CATA question format requires that respondents only check those that they believe apply (the “yes” response) [24]. Sudman [1] and Smyth et al. [10] suggest that respondents pay more attention, read all the items, and provide more thoughtful responses for CAS than CATA questions. CAS has also been shown to result in more detailed responses in terms of a mean number of affirmative checked (“agree” or “apply”) responses per respondent as compared to the CATA format [10,17,24]. This finding is also consistent with behavior survey data conducted in different languages and countries of residence [25]. However, most of the CATA–CAS comparison studies are public opinion surveys, with only one studying perceptions of food or food behavior. A potential issue with this forced-choice kind of questioning has been associated with acquiescence bias [10]. Acquiescence bias is a type of response bias where respondents tend to mark (or agree with) with the positive connotation for all survey questions [26]. Further, Best and Krueger [27] suggested that requiring an answer for each item on a questionnaire could frustrate respondents and could lead to a high number of partial completes as respondents quit the survey before it is completed. Nicolaas et al. [12] and Smyth et al. [10] did not find such effects, but they had reasonably short questionnaires of 8–15 questions. Typically, studies in the sensory literature have longer questionnaires [28,29] than those in general survey studies that have been conducted, which could impact findings. Jaeger et al. [18] showed that respondents found that rating each attribute was “slightly more tedious” than using the CATA format. Perhaps because of that, CAS is popular with telephone surveys but appears to be rarely used with in-person or web surveys for sensory consumer research [11,14]. 

The overall objective of the current online survey was to compare the CAS and CATA question formats, which were used to collect consumers’ motivations for eating five food items belonging to different food groups. Specific objectives were to collect and compare data in multiple (five) countries and a) compare the percentages of ” apply” (“yes” or “agree”) responses for CAS and CATA, b) establish the response ratios of CAS to CATA), c) identify the level of importance of the eating motivation constructs, and d) compare respondents’ mean survey duration, survey liking, just about right (JAR) rating questions, and completion rates for the two question formats of an eating motivation survey (EMS). 

## 2. Materials and Methods 

### 2.1. Eating Motivation Survey (EMS)

An eating motivation survey (EMS) questionnaire [9,30,31] was modified to include questions on consumers’ motivations for eating (in the original EMS) or not eating food items (added) that belong to five food groups [29]. The questionnaires were randomly assigned to respondents in either the CAS or CATA formats but not both (each respondent saw only one format). A total of 47 positive motivation items that could be categorized into 16 motivation constructs were assessed in each format of the EMS questionnaire (Table 1). Each eating motivation had 3 subscales or items except for the choice motivation that had only two subscales. The CATA and CAS questionnaires were designed so that respondents checked each of the motivation items that they agreed contributed to them eating that food (CATA) or checked either “yes” or “no” for each motivation (CAS) as to whether they believed it contributed to them eating that food. In the results and discussion sections of this paper, we use “agree” or “apply” to refer to responses for which the respondents selected motivation items (in CATA) or checked the “yes” option (in CAS). For CATA, all 47 items were presented on a single page, while, for the CAS question format, five questions were presented on each page. The number of CATA items (47) that were assessed in the current survey was not unusual. In fact, the literature shows several articles where a similar number of CATA items were evaluated [9,10,23,30]. 

The questionnaires focused on motivations for eating items from five food groups: foods rich in starch (e.g., potato and rice dishes), proteins (e.g., meat, beans), dairy, fruits, and sweet foods/desserts [30,32]. Food items fitting in these food groups and applicable to the particular country were used (Table 2). For example, in the case of starchy foods, baked potatoes were used for the USA, while paella was used for Spain and white rice was used for Brazil, China, and India. These foods were chosen based on discussions with multiple sensory scientists in each country who reviewed and discussed all the foods chosen in all countries to ensure the products represented the “concept” of the food category as much as possible for that country. Where possible, similar foods were used (e.g., white rice in three countries for “starchy foods”), but where the product was not widely consumed in that form (e.g., Spain) or not consumed in a similar form by a large portion of the population (e.g., USA), alternative products were selected that were more commonly eaten.

In addition to EMS, the online survey questionnaire also included other questions that were included in the survey timing. For example, two questions that investigated the respondents’ survey experience in terms of liking (a hedonic question) and a rating based on the length of the survey (a just about right or JAR question) were included near the end of the survey. The survey liking question and the JAR question were each placed on separate pages. The survey was written in English for the respondents in the USA and the survey was translated into Simplified Mandarin, Hindi, Spanish, and Portuguese for respondents in China, India (English also provided as an option), Spain, and Brazil, respectively. The survey translation process used a variation of the translation, review, adjudication, retesting, and documentation (TRAPD) approach [33,34]. The full procedure for the survey methods, including translation, and the surveys in all five languages have been published previously [29]. 

This online survey was designed following a protocol for research with human subjects (IRB #7297.2) that was approved by the designated review board at Kansas State University, Manhattan, KS, USA.

### 2.2. Respondents and Recruitment 

Respondents in five countries were recruited by Qualtrics, Provo, UT, USA using its or its partners’ existing databases. Using the Qualtrics survey software, one format of the survey questionnaire with CAS questions and another with CATA questions were assigned randomly to 400+ respondents per country (*N*~200 per questionnaire per country) [29]. Respondents were required to be 18 or older and then were recruited to fill demographic quotas of age and gender for each questionnaire format (CAS and CATA). Four age groups (*n* = 50+ per age group) were used in this study: Generation Z (born in the years 1995 to 2001), Millennials (born in the years 1980 to 1994), Generation X (born in the years 1965 to 1979) and Baby Boomers or Boomers (born in the years 1944 to 1964). For each age group, 50% were female and 50% were male. Once the required number of completed responses for a particular quota was filled, newly qualified respondents (for the filled quotas) were discontinued from completing the EMS. Other demographic data that were collected for informational purposes included respondents’ level of education, number of adults, and number of children in the households (Table 3). 

### 2.3. Data Analysis

#### 2.3.1. Comparison of Percentages of “Agree” Responses

The percentages of CAS and CATA “agree” responses for each food group in each of the five countries was calculated. Percentages were used because the possible number of ticks/checks varied depending on how many people ate that particular food in a particular country and the number of subscales in the eating motivation category. Chi-square tests were used to compare significant differences in proportions between CATA and CAS. 

#### 2.3.2. Establishment of Ratios of CAS to CATA and Standard Indices for CAS and CATA

The ratios of percentages of “agree” responses for CAS to CATA were calculated to determine if the ratio of responses varied or remained the same between the two methods. Similarly, a “standard index of importance” (SII) of responses was determined for all motivation categories versus liking within CATA or CAS. This index value shows the proportion of the number of “agree” responses for any motivation to “agree” responses for the liking motivation, which has been shown in prior studies to be the highest motivation on average [9,30]. Using liking as the comparison index factor (the denominator in the proportion calculation) within each food group, country, and consumer demographic segment allows a within-sample “variable” to be used to adjust all comparisons and put them on a similar “scale” (typically 0–1.0). Note, it is possible to exceed 1.0 when a motivation exceeds liking in importance for a group of people, although this rarely happened. Put simply, the SII is 1.0 when the “agree” responses for any motivation are equal to the “agree” responses for liking within that method for that group of respondents. Similarly, the SII would be 0.5 when a motivation response is half the number compared to liking and so forth. The index was created using the principles espoused by creators of other indices for psychological phenomena that must be compared across various segments but can vary in response behavior across segments [35,36]. If the CAS and CATA formats were assessing the same behavioral patterns of consumers, then the SII values for CAS and CATA for the different motivation constructs would be similar or relatively close. However, if the SII values for the two formats were different, this would indicate that the questions from the two formats were interpreted, processed, and answered differently by the respondents. Major differences in standard index values for motivations within CATA or CAS would suggest that the results of the two methods likely would provide different information to the researchers. Such findings would suggest that CATA and CAS, for various reasons, do not measure the same psychological phenomena or, at a minimum, the results would be interpreted differently.

#### 2.3.3. Identification of the Level of Importance for Motivation Constructs

Based on the percentages of “agree” responses for the CAS and CATA question formats of the online survey, ranking was used to identify the top five motivation constructs for each food group in each country.

#### 2.3.4. Comparison of Question Format Completion Rates, Mean Survey Duration, Survey Liking, and JAR Rating 

Analysis of variance (ANOVA) was conducted to assess the effect of survey format on survey liking, mean duration, and respondent JAR for EMS in each location. Percentages of completion rates for each of the two question formats were also calculated. 

All analyses were run using XLSTAT (version 2020.1, AddinSoft, New York, NY, USA).

## 3. Results and Discussion

### 3.1. Percentages of “Agree” Responses for CAS and CATA

The CAS and the CATA question formats collected significantly different (*p* ≤ 0.01) percentages of “agree” responses for each of the 16 positive motivation constructs in all five countries for starchy foods (Table 4). Data for proteins, dairy, fruit, and sweet/dessert foods are presented in the (Table A1, Table A2, Table A3 and Table A4). The CAS format amassed a higher percentage of “agree” responses than the CATA question format, which is similar to that mentioned or found by other authors [1,10,11,12,23,24]. For example, baked potatoes (a starchy food) in the USA (Table 4) received more “agree” responses for all of the eating motivation constructs when CAS was used as compared to when the CATA question format was used. A case in point, for the USA, 5% and 44% of respondents who completed the CATA question format identified choice limitation and liking, respectively, as important motivations for eating baked potatoes. Conversely, the percentage of responses that identified choice limitation and liking as important eating motivations was more than twice (25% and 92%, respectively) for people who answered the CAS format of the survey in the same country. Similar cases were observed for the other four countries (Brazil, China, India, and Spain) and for the other four food groups (protein-rich foods, dairy foods, fruit, and desserts). 

The large difference in the percentage of respondents who found a motivation “important” in CAS or CATA could be the result of several factors. Based on various survey theories, the higher proportion of unchecked items for the CATA format could be explained by respondents who tend to want to hurry through questionnaires and, thus, only focus on checking a few items, perhaps only the attributes or statements with the highest priority to them. Similarly, the lack of attention could also lead to checking only the first few items and not spending time reading the rest of the items but moving on to the next question after they felt they had satisfied the question requirement. Because the statement items were randomized for each respondent, the bias would be randomized throughout the test, but still influences the number of responses over all consumers. According to Jaeger et al. [21] and Smyth et al. [12], the smaller percentage of “agree” responses for CATA could partly be explained by respondents who missed them as they read the list of 47 items and it is also possible that respondents who were unsure whether an item was applicable did not mark that statement. 

Conversely, the higher percentage of responses for CAS could be ascribed to the fact that the CAS question format required a response for each of the 47 eating motivations items, which was not the case for the CATA question format. Some authors suggest that the percentage of forced-choice scores (e.g., CAS) could be high if respondents choose the “yes” or “agree” category to avoid actively saying they “disagree” with any item or statement. This reasoning suggests that requiring respondents to provide either a “yes” or a “no” for each item could cause some respondents to lean towards giving a “yes” or “agree” response. This potential acquiescence bias has been noted by sensory researchers for some populations and is sometimes referred to as a “politeness” bias [37,38,39]. However, more recent studies have not shown such “politeness” effects [40,41]. Further, if the bias were true for this set of consumers and questions, we would expect to see consistently high percentages for CAS across all products and motivations within a country. We did not. The range of percentages for CAS “agree” responses is quite large, e.g., for the starch-rich food group, the CAS percentages range from 4% to 98% for Spain and 20% to 80% for India. For CATA, the range is smaller, from 0% to 63% for Spain and 2% to 33% for China. For all food categories, there are CAS “agree” responses greater than 90% and less than 10%, suggesting that respondents were not simply checking “agree” to be polite or checking in some random way. 

The lower percentage of “agree” responses for the CATA format appears much more likely to be explained by a portion of respondents who paid lower attention to each statement and simply selected the “agree” option more randomly and chose fewer options because of that. This is supported by the fact that although less-used motivations tended to be used less in both methods, they were used so infrequently in CATA that they did not differentiate among themselves. This was not true for CAS data. For example, the least-used constructs for dairy foods generally were affect regulation, social norms, social image, visual appeal, and sociability. Within a country, the frequency of the use of and range for these attributes using the CATA format was less than 10% and 5%, respectively (except India, where it was 12% and 8% percentage, respectively). These attributes were neither discriminating nor helping to understand differences among motivations for eating by individuals using the CATA format. In comparison, these same motivations for dairy foods using the CAS format received a low of 10% to a high of 60% use (depending on the country), with a range of scores 24% points within any single country. Clearly, they were not only used more frequently in the CAS format but were also not contributing the same degree of explanation and discrimination of eating behaviors.

### 3.2. Response Ratios (CAS: CATA, Standard Index of Importance (SII))

Similar to prior studies [9,42], liking was almost always found to be peoples’ greatest motivation (higher percentage of “agree” responses overall) for eating items from the five food groups in the five countries (Table 4 and Table A1, Table A2, Table A3 and Table A4). This did not change regardless of whether CAS or CATA was used for measurement in the survey. However, the importance of other constructs did change depending on the method of measurement used, CAS or CATA. This indicated that the detail of the data collected using CAS and that collected using CATA was different. In addition to the higher percentages of “agree” responses for CAS as compared to CATA, authors noticed that the ratio of CAS to CATA “agree” responses for each of the five food groups was not only greater than one but that it also varied largely depending on the particular motivation construct that was being assessed (Table 5, Table A5, Table A6, Table A7 and Table A8). For example, the construct “health” was chosen at a higher frequency in CAS than CATA, suggesting that it may be more important than the CATA data imply. It is possible that either the CAS questions overestimated the level of importance of the health construct or that the CATA questions underestimated the level of importance of the same construct to the respondents. Further, and perhaps even more pertinent, the importance of the health construct changed (depending on the country) among the various food types when using CAS compared to CATA data. In the US, the importance of health in the dairy category (Table 5) increased 6-fold using CAS, whereas it only increased 2-fold for the fruit category (Table A5). These ratios were much more similar, approximately a 2-fold increase for both dairy and fruits, for the other countries in the study, but varied for other categories. In another example, the construct “traditional eating” in China showed large differences in importance for all food groups. However, the effect was much less for the starchy foods category (Table A6) than the protein food category (Table A7), a 3.4-fold vs. 6.2-fold increase, respectively. These changes were also noted for all other countries, but the differences among food groups were less with Brazil showing almost no variation among food groups. Quite large differences among CAS and CATA were noted for some other motivations, but those typically involved motivations where consumers actually chose that motivation infrequently in CATA. These findings point out a research gap for the validation of the CAS and CATA results using qualitative studies such as focus groups with respective populations to determine the level of accuracy that each question format provides. 

Another way to look at the importance of each construct is to compare its SII. This index, which shows how each construct or attribute compares in importance to the most important construct or attribute (in this case liking), is also shown for each of the five food groups for the five countries in Table 5, and Table A5, Table A6, Table A7 and Table A8. If the relative importance of each attribute is the same for the two methods, then the SII should be the same between the methods for each construct in each country. This was not the case in this study. Although we might expect some random variation among these values, the *difference* (SII:CAS minus SII:CATA) in the SII index ranges from 0.16 for the USA starch foods category, to 0.82 for that same category in China. This low SII index (0.16) shows some differences among methods but might be reasonably consistent, but the larger one clearly shows a large difference in information provided by the two methods may not be the same. Overall, the difference in the range of SII values within a country and food category is approximately 0.3, which would seem to indicate that the variation is enough to potentially impact subsequent data analysis and interpretation.

The variation in both percentage and index data is critically important because it shows that CAS and CATA are not necessarily providing the same information to researchers. If that is the case, researchers could infer different conclusions using the two methods. It is not a simple matter of getting more “agree” responses using the CAS format, it is a matter of getting both more and different percentages of “agree” responses depending (in this study) on the food category and the motivation. Thus, the suggestion that the primary difference in CAS and CATA is that people simply choose fewer statements or attributes when using CATA as compared to when using CAS is incorrect. This difference in scoring behavior between the two methods is a major issue that requires further investigation.

### 3.3. Level of Importance of Consumer Eating Motivations Based on CAS vs. CATA

To determine whether information from the two methods provides different interpretations would require a complete analysis of each country’s data and publication of multiple maps and interpretations for each country. However, an examination of the major themes presented by each set of data can be gained by examining the relative positioning of the constructs consumers chose in the CAS and CATA studies (Table 6). Overall, the top five eating motivations were reasonably similar within a country within a food between CAS and CATA. The top motivation, which was liking for almost all foods and countries, was common for both CAS and CATA. Similarly, habits, need/hunger, pleasure, and health typically make up the top five constructs for eating behavior across most foods and countries regardless of whether CAS or CATA is used. Convenience, traditional eating, natural concerns, weight control, visual appeal, and sociability also appeared in various lists depending on the country, the food, and the method used for testing. In general, for so-called Western cultures (USA, Spain, and Brazil), the similarities between CAS and CATA data for the main constructs were similar, with some slight variation in their rankings based on percentage agreement with the statements. This was less true for India and China, where differences in the order of importance of the constructs and differences in which constructs were chosen were more frequent. This suggests that the “big picture” information may change, but not drastically, depending on the method and, in some cases, the cultural use of the two methods. 

However, moderately used motivations (e.g., weight control, visual appeal, sociability, choice limitation, and social image) were much more likely to change in their ranked importance between CAS and CATA data within a food category within a country. For example, weight control was more likely to have a higher SII index for CATA than CAS data, pushing it into the “top 5” motivations, suggesting that it had greater importance when using that method. 

Another potential way to look at the data is to compare it to prior studies using the same basic eating motivation survey. The most similar direct comparison (same CATA questionnaire used in the USA) [30] investigated food groups among people living in the USA. These findings suggested that all foods were eaten primarily because of liking. Need and hunger factored highly into meats, starchy foods, and dairy, with health important to dairy and fruit consumption. Habits for protein foods, convenience for starchy foods, and weight control for fruits were the other highest-ranking motivations. In addition to liking, the sweets category in that study was most influenced by pleasure and choice limitation. In the current survey, CATA findings for the USA showed some similarities and several differences in how respondents perceived the level of importance of each motivation construct for the five food groups that were tested. In both studies, liking was predominant and need and hunger often was key. Other constructs such as convenience, weight control, or health moved around somewhat or disappeared from the top set of motivations in one study or the other for some food groups. Other research in the USA [43] using the same set of constructs (except for choice limitation) and using a scale instead of a CATA measure found that liking, need and hunger, and habit were the three most important constructs with health, convenience, and pleasure next. These authors did not separate foods by groups. In that same study, results from India showed that liking, pleasure, need and hunger, and health were most important. This is somewhat different than the CATA findings from this study, which never showed pleasure in the top five of importance for India except for desserts. In Brazil [44], a study similar to the one from India showed that liking, habits, and need and hunger were the three primary constructs used by consumers to make food choices. Liking and habits were common findings in both that and our study. However, the current study with CATA data for India does not show need and hunger as one of the top three factors for any food, although it does tie for fourth place with fruit and dairy foods. In the CATA portion of this study, factors such as health, traditional eating, and natural concerns are more important. These differences to other studies show that CATA data find similar, but not the same information. These differences could be the result of the question format(s), e.g., the CATA format, or other issues including differences in the populations tested within each country, and differences in the recency of testing (although they were all completed within 5 years of each other). 

The differences in response ratio for CAS and CATA in the current study suggest that motivations for eating foods could be shown to be more or less important depending on whether the CAS or CATA question formats were used in online consumer surveys [11]. This would be critical in how consumer data is interpreted and what decisions would be made thereafter. For example, product developers and sensory scientists usually use such data to identify product sensory attributes that drive consumer acceptance to guide product reformulations and product-line extensions. Sensory scientists may apply the CATA and CAS question formats in studies for product claim substantiation and studies that seek to understand more product consumer psychographics. Nutritionists and dietitians use these two question formats in baseline surveys when designing health and nutrition interventions and services for communities. However, without knowing which of the CATA or CAS question formats collects more accurate data, it may be difficult to recommend the use of one question format over the other in future online surveys. Information and interpretation based on these online surveys must be accurate if the consumer researchers are to attain their desired objective. This calls for careful consideration by consumer researchers when choosing to use either CATA or CAS in their online consumer surveys [11]. 

### 3.4. Mean Duration, Just About Right (JAR) Rating, Consumer Liking, and Completion Rates for CAS and CATA

The average mean duration to complete the CAS and CATA questions were found to be significantly different for countries such as the USA, China, and Spain. For these three countries, it took the respondents significantly longer to complete the CAS questionnaire than the CATA questionnaire (Table 7). Smyth et al. [10] attributed the longer times taken to complete the CAS questions to the demand for deeper thought processes, as stated by Sudman and bardburn [1], and also the time consumed when selecting the disagree or “no” options, which was not the case for the CATA format of the survey. There was, however, no difference in the mean time required to complete both the CAS and CATA questions for respondents in Brazil and India. Both of these countries took longer to complete the questionnaire than respondents in other countries.

Respondents in the USA, Spain, China, and Brazil rated the CAS format as a little too long and the CATA format as just about right (Table 8). This partly explained the significantly higher scores for survey liking that the respondents in these countries expressed for the CATA format when they were asked about how they felt about the survey experience (Table 9). Although respondents in India also liked the CATA format more than the CAS format of the survey, they did not have a significant difference in the length of the two question formats of the survey. 

**Figure 1:**
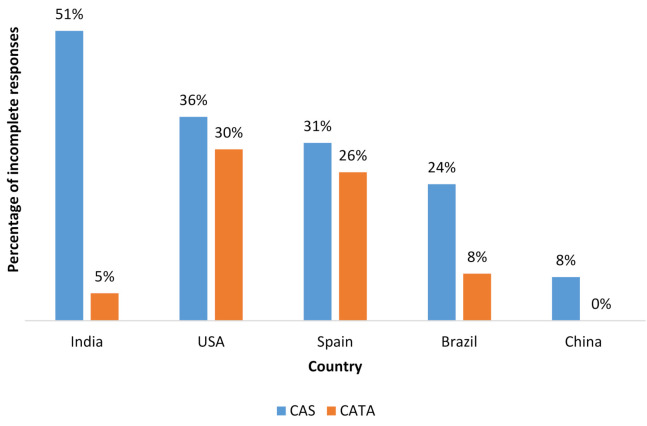
Percentage of incomplete responses for CAS and CATA per country. Incompete questionnaires were not accepted or used and were not counted in the approximately 200 responses received per country per questionnaire type.

The CAS version of the survey had a higher percentage of incomplete responses as compared to the CATA questionnaire (Figure 1). This was highlighted in India where the rate of survey completion for CAS was less than 50% as compared to the 95% completion rate for the CATA questionnaire in the same country. Such information must be considered as part of the overall planning for studies. However, completion rates and consumer liking of questionnaires should never be used as a reason to change a questionnaire format to one that collects data if the format will provide less or lower quality data and information.

### 3.5. Survey Limitations

This study has several limitations that must be considered when comparing the methods, food groups, and countries. First, except for the fruits and vegetables food group, where the banana was tested in all five countries, some different food items were used to test the other food groups in the countries. Differences in products chosen, such as potato for the USA and rice for all other countries, could have affected the reliability of the findings between countries in the online survey. However, specific differences among countries were not the focus of this study and the use of commonly eaten foods in each country likely was more critical to this study than a consistent food for each country that few people in a country might eat. Consumer researchers designing future similar studies may choose to have half of the products the same for all locations and the other half custom picked to match the individual locations that are being investigated. Such information might increase the cross-country comparison reliability, but would also double the size of the sample needed. 

A more meaningful limitation is that all respondents who completed this survey in the five countries were literate and had access to the internet. This indicates that parts of the population(s) who were illiterate or had no access to the internet at the time of fielding were not included. Access is a common problem with surveys and each survey type has limitations related to its method of fielding. In this study, although the population was technically “national”, most databases for online surveys have a preponderance of consumers with higher than average access to the internet. Survey respondents are also literate, have time and are willing to take an online survey, and can be distracted by other influences during the testing, which can slow them down or result in them becoming more frustrated with surveys that are more time consuming or more difficult to complete. Similar future international research surveys could be extended to include the responses of people who may not have access to the internet or be conducted in areas with more limited access to testing. However, survey location is a limitation of in-person surveys that usually are conducted in only one or a few locations, which provides a limited sample. For people who are unable to read, the survey can be read to them, either by telephone (if available) or in person. Such in-person, one-on-one testing has been reported, particularly in countries where literacy rates are low, but typically is not used for most survey research because of the large increase in resources required for such testing. 

## 4. Conclusions

This online survey confirmed that the CAS question format provided more “apply” responses per attribute construct as compared to the CATA questionnaire format. Further, the response ratio of CATA to CAS responses was found to be different for each motivation construct, each food group, and for each country. This suggested that the level of importance that was accorded by the respondents to each motivation construct though similar in a few cases differed largely depending on whether the CAS or CATA question format was used in the online survey. The SII varied greatly within the CAS format and varied less for the CATA format, implying that the CAS format was much more discriminating among the motivation constructs than the CATA format. Although the overall “big picture” of the main constructs may appear similar when examining the ratios, the constructs that were not part of the top five appear to vary more, which alludes to differences in interpretation that may occur when the two methods are used and detailed information is needed. 

This study suggests that more research is needed before consumer researchers can use the CATA and CAS formats interchangeably in their online survey questionnaires. The work highlights a need for additional research to understand the reasons for the large discrepancies in responses in the two methods. Such research is needed to determine which method, if either, provides a more accurate assessment of consumers’ determination of the importance or presence of characteristics such as motivations for eating to more effectively guide consumer researchers on when best to use either question format in future studies. 

## Figures and Tables

**Table 1:** The 16 eating motivation constructs (**bold**) and their corresponding ***positive*** subitems that were used in the eating motivation survey (EMS) (adapted from [9,31]).

**Liking**	**Sociability**
Because it tastes good	Because it is social
Because I like it	So that I can spend time with other people
Because I have an appetite for it	Because it makes social gatherings more comfortable
**Habits**	**Price**
Because I usually eat it	Because it is inexpensive
Because I am familiar with it	Because it is on sale
Because I’m accustomed to eating it	Because I don’t want to spend any more money
**Need and Hunger**	**Visual Appeal**
Because I’m hungry	Because it spontaneously appeals to me
Because it is pleasantly filling	Because the presentation is appealing (e.g., packaging)
Because I need energy	Because I recognize it from advertisements or have seen it on TV
**Health**	**Weight Control**
Because it is healthy	Because it is low in calories
To maintain a balanced diet	Because it is low in fat
Because it keeps me in shape (e.g., energetic, motivated)	Because I watch my weight
**Convenience**	**Affect Regulation**
Because it is quick to prepare	Because I am sad
Because it is the most convenient	Because I feel lonely
Because it is easy to prepare	Because I am frustrated
**Pleasure**	**Social Norms**
Because I enjoy it	Because I am supposed to eat it
In order to indulge myself	Because it would be impolite not to eat it
In order to reward myself	To avoid disappointing someone who is trying to make me happy
**Traditional Eating**	**Social Image**
Because I grew up with it	Because others like it
Because it belongs to certain situations	Because it is trendy
Out of traditions (e.g., family traditions, special occasions)	Because it makes me look good in front of others
**Natural Concerns**	**Choice Limitation**
Because it is organic	I want to eat it every day
Because it contains no harmful substances	Because it is the only choice
Because it is natural (e.g., not genetically modified)	

**Table 2:** Food items that were used for each food group in each of the five countries.

Countries	Starchy Foods (Carbohydrates)	Proteins Foods (Meat, Fish, and Eggs)	Milk and Dairy Foods	Fruit and Vegetables	Desserts (Fats and Sugars)
Brazil	White rice	Feijao	Milk	Bananas	Brigadeiro
China	White rice	Red braised pork belly	Soy milk	Bananas	Pan-fried red bean paste cakes
India	White rice	Toor dal	Milk	Bananas	Gulab jamun
Spain	Paella	Jamón serrano	Milk	Bananas	Turrón
USA	Baked potato	Hamburger	Cheese	Bananas	Chocolate cake with frosting

**Table 3:** Overview of demographic segmentation of respondents who completed the CAS and CATA question formats of the EMS in all five countries ^1^.

	USA		Brazil		Spain		India		China	
	CAS	CATA	CAS	CATA	CAS	CATA	CAS	CATA	CAS	CATA
**Gender**										
Men	105	106	100	100	107	106	132	120	102	103
Women	107	108	100	100	107	107	130	137	103	108
**Age Group**										
Boomers	54	54	50	50	52	52	55	57	53	52
Generation X	53	53	50	50	54	54	76	70	51	53
Generation Y	53	54	50	50	54	54	54	65	49	52
Generation Z	52	53	50	50	54	53	77	65	52	54
**Education Level**										
Primary school or less	1	4	6	4	7	7	13	14	10	10
High school	114	94	87	94	81	99	52	42	67	67
College or university	97	116	107	102	126	107	197	201	128	134
**Adults in Household**										
One	50	62	18	19	17	15	12	16	6	4
Two or more	162	152	182	181	197	198	250	241	199	207
**Children in Household**										
None	143	133	95	102	117	110	106	119	80	91
One or more	69	81	105	98	97	103	156	138	125	120

^1^ Number of respondents.

**Table 4:** Percentage of “agree” responses for CAS and CATA for all five countries for the respective starch-rich foods. *

	USA		Brazil		Spain		India		China	
	CAS	CATA	CAS	CATA	CAS	CATA	CAS	CATA	CAS	CATA
Liking	92	44	94	48	98	63	80	31	76	21
Habits	68	22	89	43	74	33	82	25	84	33
Need & Hunger	71	23	75	26	77	23	73	15	78	19
Health	46	12	54	20	51	18	74	23	63	17
Convenience	64	25	63	27	26	9	72	22	69	18
Pleasure	64	23	53	13	79	32	74	20	44	7
Traditional eating	47	14	43	24	71	28	72	23	63	19
Natural concerns	46	12	50	12	49	9	74	19	63	13
Sociability	22	4	32	9	52	13	52	9	41	5
Price	43	13	29	8	15	3	43	7	35	6
Visual appearance	38	8	15	3	36	10	56	11	40	4
Weight control	34	8	32	12	15	4	55	13	42	9
Affect regulation	14	3	3	1	4	0	28	3	15	2
Social norms	18	4	19	5	17	2	39	5	37	8
Social image	23	4	8	2	15	4	38	5	32	3
Choice limitation	25	5	43	11	20	2	51	12	47	9

* All percentages within a country and construct (e.g., CAS vs. CATA for liking in the USA) were significantly different (*p* ≤ 0.01).

**Table 5:** Ratios of CAS “agree” responses to CATA “agree” responses and standard indices of importance for CAS and CATA “agree” responses for each motivation construct to the liking construct for dairy foods in all five countries.

	USA			Brazil			Spain			India			China		
	R	S	T	R	S	T	R	S	T	R	S	T	R	S	T
Liking	2.1	1.00	1.00	1.9	1.00	1.00	1.7	1.00	1.00	2.7	1.00	1.00	3.4	1.00	1.00
Habits	3.1	0.87	0.59	2.3	0.86	0.71	2.4	0.95	0.67	3.9	1.03	0.71	3.4	0.94	0.95
Need & Hunger	3.6	0.72	0.41	2.8	0.69	0.46	3.2	0.76	0.39	3.6	0.95	0.71	5.8	0.69	0.41
Health	6.1	0.41	0.14	2.2	0.65	0.55	1.9	0.69	0.59	2.4	1.01	1.14	2.9	0.98	1.15
Convenience	3.8	0.85	0.47	2.8	0.71	0.47	2.9	0.78	0.45	4.9	0.92	0.50	4.9	0.79	0.56
Pleasure	2.8	0.76	0.57	3.6	0.59	0.31	2.8	0.71	0.42	4.4	0.97	0.58	5.5	0.60	0.37
Traditional eating	3.2	0.63	0.41	2.1	0.43	0.38	2.8	0.66	0.39	3.4	0.90	0.70	4.6	0.63	0.47
Natural concern	4.9	0.36	0.15	4.9	0.46	0.18	3.4	0.54	0.27	3.0	1.00	0.89	5.6	0.86	0.52
Sociability	5.1	0.29	0.12	4.4	0.23	0.10	8.9	0.19	0.04	8.9	0.69	0.21	7.3	0.42	0.20
Price	3.6	0.48	0.27	3.9	0.25	0.12	5.3	0.29	0.09	5.6	0.59	0.28	3.9	0.47	0.41
Visual appeal	6.5	0.45	0.14	9.3	0.17	0.03	9.7	0.30	0.05	4.8	0.77	0.43	8.0	0.56	0.24
Weight control	4.0	0.27	0.14	4.8	0.32	0.12	2.7	0.34	0.21	5.1	0.76	0.40	4.3	0.76	0.61
Affect regulation	4.9	0.19	0.08	5.0	0.03	0.01	27.0	0.11	0.01	9.8	0.47	0.13	10.1	0.24	0.08
Social norm	8.2	0.26	0.06	3.4	0.21	0.12	3.5	0.23	0.11	7.1	0.59	0.22	7.3	0.45	0.21
Social image	4.8	0.25	0.11	2.5	0.05	0.04	9.6	0.15	0.03	6.2	0.57	0.25	4.7	0.40	0.30
Choice limitation	4.2	0.25	0.12	3.3	0.29	0.17	3.4	0.29	0.14	4.1	0.52	0.34	6.1	0.31	0.17

R = ratio of CAS “agree” responses to CATA “agree” responses, S = standard index of CAS “agree” responses for each construct to liking, and T = standard index of CATA “agree” responses for each construct to liking.

**Table 6:** Rank of the top five motivation constructs based on percentages within each food group and each country for both CAS and CATA ^1^.

	USA		Brazil		Spain		India		China	
	CAS	CATA	CAS	CATA	CAS	CATA	CAS	CATA	CAS	CATA
**Starchy foods**										
Liking										
Habits										
Need/Hunger										
Pleasure										
Convenience										
Health										
Traditional Eating										
Natural Concerns										
**Protein foods**										
Liking										
Habits										
Need/Hunger										
Pleasure										
Convenience										
Health										
Traditional Eating										
Natural Concerns										
Visual Appeal.										
**Dairy foods**										
Liking										
Habits										
Need/Hunger										
Pleasure										
Convenience										
Health										
Traditional Eating										
Nauralt. Concerns										
Weight Control										
**Fruit**										
Liking										
Habits										
Need/Hunger										
Pleasure										
Convenience										
Health										
Natural Concerns										
Weight Control										
**Desserts**										
Liking										
Habits										
Need/Hunger										
Pleasure										
Convenience										
Traditional Eating										
Visual Appeal.										
Sociability										

^1^ Rank color codes for the top five motivation constructs: purple = first position, red = second position, yellow = third position, green = fourth position, and blue = fifth position.

**Table 7:** Means ^1^ and *p*-values for the survey mean duration for CAS and CATA per country.

	Brazil	China	India	Spain	USA
CAS	33.6	24.5	32.9	23.6	20.9
CATA	32.9	14.7	28.0	17.7	12.4
*p*-value	0.7576	<0.0001 *	0.3718	0.0044 *	<0.0001 *

^1^ Mean duration in minutes; * *p*-values with an asterisk indicate that CAS and CATA LS means differed significantly (*p* ≤ 0.05).

**Table 8:** Means ^†^ and *p*-values for just about right ratings for CAS and CATA per country.

	Brazil	China	India	Spain	USA
CAS	4.9	4.8	3.6	5.1	4.6
CATA	4.3	4.3	3.7	4.3	4.2
*p*-values	<0.0001 *	<0.0001 *	0.960	<0.0001 *	<0.0001 *

^†^ Seven-point scale; 1 = much too short, 4 = just about right (JAR), and 7 = much too long; * *p*-values with an asterisk indicate that CAS and CATA LS means differed significantly (*p* ≤ 0.05).

**Table 9:** Means ^†^ and *p*-values for survey liking for CAS and CATA per country.

	Brazil	China	India	Spain	USA
CAS	4.1	3.7	4.1	3.9	3.8
CATA	4.4	3.9	4.3	4.2	4.1
*p*-value	<0.0001 *	0.003 *	0.024	<0.0001 *	0.000 *

^†^ Five-point scale; 1 = I hated taking it, 3 = I have no feelings either way, and 5 = I liked it a lot; * *p*-values with an asterisk indicate that CAS and CATA LS means differed significantly (*p* ≤ 0.05).

## Appendix A

**Table A1:** Percentage of “agree” responses for CAS and CATA for all five countries for the respective protein-rich foods.*

	USA		Brazil		Spain		India		China	
	CAS	CATA	CAS	CATA	CAS	CATA	CAS	CATA	CAS	CATA
Liking	93	49	97	56	95	70	82	35	86	31
Habits	74	25	89	43	84	37	82	25	57	16
Need/Hunger	74	25	79	30	75	27	72	13	65	19
Health	26	6	62	30	58	19	81	31	37	9
Convenience	68	21	42	15	64	23	72	19	29	4
Pleasure	74	28	52	16	78	44	72	17	62	17
Traditional. eating	57	15	43	21	74	25	69	22	46	7
Natural. concern	31	6	52	12	53	15	77	23	42	5
Sociability	34	8	29	8	46	11	53	8	38	6
Price	45	12	22	5	15	2	44	8	24	3
Visual appeal	51	6	17	4	41	9	57	13	50	8
Weight control	20	4	30	11	32	6	60	18	20	3
Affect regulation	16	3	4	0	9	1	32	5	18	2
Social norm	21	4	25	8	19	2	43	6	33	4
Social image	24	5	7	2	18	3	41	6	28	3
Choice Limitation	28	6	44	17	31	9	51	12	24	4

* All percentages within a country and construct (e.g., CAS vs. CATA for liking in the USA) were significantly different (*p* ≤ 0.01).

**Table A2:** Percentage of “agree” responses for CAS and CATA for all five countries for the respective milk and dairy foods. *

	USA		Brazil		Spain		India		China	
	CAS	CATA	CAS	CATA	CAS	CATA	CAS	CATA	CAS	CATA
Liking	84	41	95	51	92	55	78	29	76	22
Habits	73	24	82	36	87	37	80	21	72	21
Need/Hunger	61	17	66	23	70	22	74	21	53	9
Health	34	6	62	28	63	33	79	33	75	26
Convenience	71	19	67	24	72	25	72	15	60	12
Pleasure	64	23	57	16	65	23	75	17	46	8
Traditional eating	53	17	41	19	61	22	70	20	48	10
Natural concern	31	6	44	9	50	15	78	26	66	12
Sociability	25	5	22	5	18	2	54	6	32	4
Price	40	11	24	6	27	5	46	8	36	9
Visual appeal	38	6	16	2	28	3	60	12	43	5
Weight control	23	6	30	6	32	12	59	12	58	14
Affect reg.	16	3	3	1	10	0	37	4	19	2
Social norm	22	3	20	6	21	6	46	6	35	5
Social image	21	4	5	2	14	1	45	7	31	7
Choice Limitation	32	8	42	13	39	12	61	15	35	6

* All percentages within a country and construct (e.g., CAS vs. CATA for liking in the USA) were significantly different (*p* ≤ 0.01).

**Table A3:** Percentage of “agree” responses for CAS and CATA for all five countries for fruit. *

	USA		Brazil		Spain		India		China	
	CAS	CATA	CAS	CATA	CAS	CATA	CAS	CATA	CAS	CATA
Liking	89	43	97	58	91	62	80	28	76	27
Habits	74	22	81	39	80	31	77	21	70	20
Need/Hunger	72	25	79	35	83	36	76	21	53	10
Health	63	28	74	40	73	41	79	32	66	25
Convenience	77	21	65	18	75	23	63	10	68	16
Pleasure	62	22	58	18	72	27	74	17	47	11
Traditional eating	38	14	38	18	52	14	64	15	39	8
Natural concerns	52	17	66	22	64	24	74	24	64	13
Sociability	19	5	20	3	18	2	53	8	33	4
Price	48	13	32	15	27	5	54	12	34	8
Visual appeal	34	6	19	3	34	5	57	12	44	5
Weight control	48	13	52	22	36	14	63	16	54	13
Affect regulation	15	4	7	0	11	1	38	5	19	3
Social norm	24	6	25	9	21	4	46	6	38	5
Social image	23	7	8	2	13	1	44	7	30	7
Choice Limitation	31	10	38	13	30	11	56	10	30	6

* All percentages within a country and construct (e.g., CAS vs. CATA for liking in the USA) were significantly different (*p* ≤ 0.01).

**Table A4:** Percentage of “agree” responses for CAS and CATA for all five countries for the respective desserts. *

	USA		Brazil		Spain		India		China	
	CAS	CATA	CAS	CATA	CAS	CATA	CAS	CATA	CAS	CATA
Liking	86	41	97	60	92	51	76	35	74	25
Habits	61	15	59	20	68	22	73	19	56	15
Need/Hunger	51	16	44	15	55	16	64	15	66	13
Health	19	3	14	3	28	4	54	11	38	8
Convenience	35	7	44	13	49	8	54	10	46	9
Pleasure	79	38	67	27	83	37	76	21	56	10
Traditional eating	49	16	39	15	75	38	66	13	49	10
Natural concerns	25	5	16	2	38	7	54	10	44	5
Sociability	34	7	47	15	48	16	54	12	31	5
Price	25	5	22	4	15	2	48	9	40	8
Visual appeal	49	8	34	9	45	10	65	15	55	6
Weight control	15	2	7	1	17	1	48	8	34	3
Affect regulation	22	5	27	5	16	2	40	7	31	4
Social norm	25	6	16	5	21	3	47	6	41	4
Social image	25	7	11	3	19	4	50	9	31	8
Choice Limitation	26	6	21	6	17	3	50	12	35	6

* All percentages within a country and construct (e.g., CAS vs. CATA for liking in the USA) were significantly different (*p* ≤ 0.01).

**Table A5:** Ratios of CAS “agree” responses to CATA “agree” responses and standard indices of importance for CAS and CATA “agree” responses for each motivation construct to the liking construct for fruits and vegetables in all five countries.

	USA			Brazil			Spain			India			China		
	R	S	T	R	S	T	R	S	T	R	S	T	R	S	T
Liking	2.1	1.00	1.00	1.7	1.00	1.00	1.5	1.00	1.00	2.9	1.00	1.00	2.8	1.00	1.00
Habits	3.3	0.83	0.52	2.1	0.84	0.67	2.6	0.88	0.50	3.7	0.97	0.74	3.6	0.93	0.73
Need/Hunger	2.9	0.81	0.57	2.3	0.82	0.60	2.3	0.91	0.58	3.7	0.96	0.74	5.5	0.70	0.35
Health	2.2	0.71	0.66	1.9	0.77	0.69	1.8	0.81	0.66	2.5	1.00	1.14	2.7	0.87	0.92
Convenience	3.6	0.86	0.49	3.5	0.67	0.32	3.2	0.82	0.38	6.3	0.79	0.36	4.3	0.90	0.59
Pleasure	2.8	0.69	0.51	3.2	0.60	0.31	2.7	0.79	0.43	4.4	0.93	0.60	4.2	0.62	0.42
Traditional eating	2.7	0.42	0.33	2.1	0.39	0.31	3.8	0.57	0.22	4.2	0.81	0.55	5.0	0.52	0.29
Natural concerns	3.1	0.59	0.38	3.0	0.68	0.38	2.6	0.70	0.39	3.1	0.93	0.86	4.8	0.84	0.49
Sociability	4.1	0.22	0.11	6.0	0.21	0.06	9.0	0.20	0.03	6.9	0.66	0.28	8.1	0.43	0.15
Price	3.6	0.54	0.31	2.1	0.33	0.26	5.5	0.29	0.08	4.3	0.68	0.45	4.1	0.45	0.31
Visual app	5.3	0.38	0.15	6.1	0.19	0.05	6.6	0.38	0.08	4.8	0.72	0.43	8.4	0.59	0.19
Weight control	3.6	0.54	0.31	2.3	0.53	0.39	2.6	0.39	0.22	4.0	0.79	0.56	4.3	0.72	0.47
Affect regulation.	3.9	0.17	0.09	18.5	0.07	0.01	8.8	0.12	0.02	7.3	0.48	0.19	7.1	0.25	0.10
Social norm	3.9	0.27	0.14	2.8	0.26	0.16	4.9	0.23	0.07	7.9	0.58	0.21	7.0	0.50	0.20
Social image	3.2	0.26	0.17	4.4	0.08	0.03	12.3	0.15	0.02	6.8	0.56	0.23	4.5	0.39	0.25
Choice Limitation	3.3	0.24	0.15	2.9	0.26	0.15	2.9	0.22	0.11	5.6	0.47	0.24	5.1	0.27	0.14

R = ratio of CAS “agree” responses to CATA “agree” responses, S = standard index of CAS “agree” responses for each construct to liking, and T = standard index of CATA “agree” responses for each construct to liking.

**Table A6:** Ratios of CAS “agree” responses to CATA “agree” responses and standard indices of importance for CAS and CATA “agree” responses for each motivation construct to the liking construct for starch-rich foods in all five countries.

	USA			Brazil			Spain			India			China		
	R	S	T	R	S	T	R	S	T	R	S	T	R	S	T
Liking	2.1	1.00	1.00	1.9	1.00	1.00	1.6	1.00	1.00	2.6	1.00	1.00	3.7	1.00	1.00
Habits	3.1	0.73	0.50	2.1	0.94	0.88	2.2	0.76	0.53	3.3	1.02	0.80	2.5	1.11	1.60
Need/Hunger	3.1	0.77	0.52	2.9	0.80	0.54	3.4	0.79	0.36	4.9	0.92	0.48	4.1	1.03	0.92
Health	3.7	0.49	0.28	2.8	0.58	0.41	2.9	0.52	0.28	3.3	0.93	0.74	3.8	0.84	0.80
Convenience	2.5	0.69	0.58	2.3	0.67	0.56	3.0	0.27	0.14	3.4	0.91	0.70	3.8	0.91	0.88
Pleasure	2.8	0.69	0.53	4.0	0.57	0.28	2.5	0.81	0.50	3.7	0.93	0.65	6.3	0.58	0.34
Traditional eating	3.4	0.51	0.32	1.8	0.46	0.49	2.5	0.73	0.45	3.1	0.90	0.76	3.4	0.83	0.90
Natural concern	3.9	0.50	0.27	4.1	0.53	0.25	5.6	0.50	0.14	3.9	0.93	0.62	4.9	0.84	0.63
Sociability	5.5	0.23	0.09	3.7	0.34	0.18	3.9	0.53	0.21	5.8	0.65	0.29	8.5	0.54	0.23
Price	3.4	0.47	0.29	3.4	0.31	0.17	5.4	0.15	0.04	6.2	0.53	0.23	6.1	0.46	0.27
Visual appeal	4.5	0.41	0.19	4.9	0.16	0.06	3.7	0.37	0.16	4.9	0.70	0.37	9.5	0.53	0.20
Weight control	4.3	0.37	0.18	2.7	0.34	0.24	4.0	0.15	0.06	4.2	0.69	0.42	4.8	0.55	0.42
Affect regulation.	5.0	0.15	0.06	4.3	0.03	0.01	24.8	0.04	0.00	8.2	0.35	0.11	6.8	0.20	0.11
Social norm	4.5	0.19	0.09	3.5	0.20	0.11	7.3	0.17	0.04	7.5	0.49	0.17	4.8	0.49	0.38
Social image	5.3	0.25	0.10	4.0	0.09	0.04	4.3	0.16	0.06	7.1	0.47	0.17	10.5	0.42	0.15
Choice Limitation	4.8	0.18	0.08	3.9	0.30	0.15	9.0	0.13	0.02	4.2	0.43	0.26	5.3	0.42	0.29

R = ratio of CAS “agree” responses to CATA “agree” responses, S = standard index of CAS “agree” responses for each construct to liking, and T = standard index of CATA “agree” responses for each construct to liking.

**Table A7:** Ratios of CAS “agree” responses to CATA “agree” responses and standard indices of importance for CAS and CATA “agree” responses for each motivation construct to the liking construct for protein-rich foods in all five countries.

	USA			Brazil			Spain			India			China		
	R	S	T	R	S	T	R	S	T	R	S	T	R	S	T
Liking	1.9	1.00	1.00	1.7	1.00	1.00	1.4	1.00	1.00	2.3	1.00	1.00	2.8	1.00	1.00
Habits	2.9	0.79	0.51	2.1	0.92	0.77	2.3	0.88	0.53	3.3	1.00	0.71	3.6	0.67	0.52
Need/Hunger	2.9	0.79	0.51	2.6	0.82	0.54	2.8	0.79	0.38	5.4	0.88	0.38	3.5	0.76	0.61
Health	4.5	0.28	0.12	2.0	0.64	0.54	3.0	0.61	0.28	2.6	0.99	0.89	4.0	0.43	0.30
Convenience	3.3	0.73	0.42	2.9	0.43	0.26	2.8	0.68	0.33	3.8	0.88	0.55	7.3	0.34	0.13
Pleasure	2.6	0.79	0.58	3.2	0.54	0.29	1.8	0.82	0.63	4.2	0.89	0.50	3.5	0.72	0.57
Traditional eating	3.7	0.62	0.31	2.0	0.44	0.38	2.9	0.78	0.36	3.2	0.85	0.62	6.2	0.54	0.24
Natural concern	4.9	0.33	0.13	4.4	0.53	0.21	3.5	0.56	0.22	3.3	0.95	0.67	8.8	0.49	0.16
Sociability	4.4	0.37	0.16	3.6	0.30	0.15	4.1	0.48	0.16	6.4	0.65	0.23	6.9	0.44	0.18
Price	3.8	0.49	0.24	4.9	0.23	0.08	8.1	0.16	0.03	5.8	0.55	0.22	6.9	0.28	0.11
Visual appeal.	8.2	0.55	0.13	4.6	0.18	0.07	4.7	0.43	0.13	4.3	0.69	0.38	6.3	0.59	0.26
Weight control	5.7	0.22	0.07	2.6	0.31	0.20	4.9	0.33	0.09	3.4	0.73	0.50	5.7	0.23	0.11
Affect regulation	5.3	0.17	0.06	11.0	0.04	0.01	13.6	0.10	0.01	5.9	0.40	0.16	10.5	0.21	0.06
Social norm	5.3	0.23	0.08	3.3	0.26	0.14	12.2	0.20	0.02	7.8	0.53	0.16	8.7	0.39	0.12
Social image	4.9	0.26	0.10	3.1	0.08	0.04	5.3	0.19	0.05	6.6	0.50	0.18	8.7	0.33	0.11
Choice Limiation	4.5	0.20	0.08	2.6	0.30	0.20	3.6	0.22	0.08	4.4	0.42	0.22	6.0	0.19	0.09

R = ratio of CAS “agree” responses to CATA “agree” responses, S = standard index of CAS “agree” responses for each construct to liking, and T = standard index of CATA “agree” responses for each construct to liking.

**Table A8:** Ratios of CAS “agree” responses to CATA “agree” responses standard indices of importance for CAS and CATA “agree” responses for each motivation construct to the liking construct for desserts in all five countries.

	USA			Brazil			Spain			India			China		
	R	S	T	R	S	T	R	S	T	R	S	T	R	S	T
Liking	2.1	1.00	1.00	1.6	1.00	1.00	1.8	1.00	1.00	2.2	1.00	1.00	3.0	1.00	1.00
Habits	4.1	0.71	0.36	2.9	0.61	0.34	3.1	0.74	0.43	3.8	0.97	0.55	3.8	0.77	0.60
Need/Hunger	3.2	0.59	0.39	3.0	0.45	0.24	3.4	0.60	0.32	4.4	0.84	0.42	5.1	0.89	0.53
Health	7.2	0.22	0.06	4.3	0.15	0.05	7.4	0.31	0.07	5.0	0.71	0.31	4.8	0.52	0.33
Convenience	5.3	0.41	0.16	3.3	0.46	0.22	6.0	0.53	0.16	5.2	0.71	0.30	5.1	0.62	0.36
Pleasure	2.1	0.92	0.92	2.5	0.69	0.44	2.2	0.91	0.73	3.6	1.01	0.61	5.5	0.75	0.41
Traditional eating	3.1	0.57	0.39	2.5	0.40	0.25	2.0	0.82	0.75	4.9	0.87	0.39	4.8	0.66	0.41
Natural concerns	5.6	0.30	0.11	7.0	0.16	0.04	5.3	0.41	0.14	5.4	0.72	0.29	9.6	0.60	0.19
Sociability	4.6	0.40	0.18	3.1	0.49	0.25	3.1	0.52	0.31	4.4	0.72	0.35	5.9	0.42	0.21
Price	5.1	0.30	0.12	5.6	0.22	0.06	7.3	0.16	0.04	5.4	0.63	0.26	4.8	0.54	0.34
Visual appeal	6.0	0.57	0.20	3.6	0.35	0.15	4.3	0.49	0.20	4.2	0.86	0.44	8.4	0.74	0.26
Weight control	7.1	0.18	0.05	5.3	0.07	0.02	33.6	0.18	0.01	5.7	0.63	0.24	11.1	0.46	0.13
Affect regulation	4.1	0.26	0.13	5.0	0.27	0.09	10.3	0.17	0.03	5.9	0.53	0.19	8.4	0.42	0.15
Social norm	4.3	0.30	0.14	3.3	0.16	0.08	7.5	0.23	0.05	7.3	0.62	0.18	10.3	0.56	0.16
Social image	3.8	0.30	0.16	3.6	0.11	0.05	4.9	0.20	0.07	5.8	0.66	0.25	4.0	0.42	0.31
Choice limitation	4.4	0.20	0.10	3.8	0.14	0.06	6.4	0.12	0.03	4.2	0.44	0.22	6.2	0.31	0.15

R = ratio of CAS “agree” responses to CATA “agree” responses, S = standard index of CAS “agree” responses for each construct to liking, and T = standard index of CATA “agree” responses for each construct to liking.
